# Homoharringtonine combined with aclarubicin and cytarabine synergistically induces apoptosis in t(8;21) leukemia cells and triggers caspase‐3‐mediated cleavage of the AML1‐ETO oncoprotein

**DOI:** 10.1002/cam4.913

**Published:** 2016-10-05

**Authors:** Jiang Cao, Hao Feng, Ning‐Ning Ding, Qing‐yun Wu, Chong Chen, Ming‐Shan Niu, Wei Chen, Ting‐Ting Qiu, Hong‐Hu Zhu, Kai‐Lin Xu

**Affiliations:** ^1^ Department of HematologyThe Affiliated Hospital of Xuzhou Medical CollegeXuZhou221002China; ^2^ Department of HematologyPeking University People's HospitalBeijing100044China

**Keywords:** Acute myeloid leukemia, AML1‐ETO, homoharringtonine combined with aclarubicin and cytarabine, synergistic

## Abstract

Homoharringtonine combined with aclarubicin and cytarabine (HAA) is a highly effective treatment for acute myeloid leukemia (AML), especially for t(8;21) AML. However, the underlying mechanisms by which HAA kills t(8;21) AML cells remain unclear. In this study, SKNO‐1 and Kasumi‐1 cells with t(8;21) were used. Compared with individual or pairwise administration of homoharringtonine, aclarubicin, or cytarabine, HAA showed the strongest inhibition of growth and induction of apoptosis in SKNO‐1 and Kasumi‐1 cells. HAA caused cleavage of the AML1‐ETO (AE) oncoprotein to form truncated AE (ΔAE). Pretreatment with the caspase‐3 inhibitor caspase‐3 inhibitor Q‐DEVD‐OPh (QDO) not only suppressed HAA‐induced apoptosis but also abrogated the cleavage of AE and generation of ΔAE. These results suggest that HAA synergistically induces apoptosis in t(8;21) leukemia cells and triggers caspase‐3‐mediated cleavage of the AML1‐ETO oncoprotein, thus providing direct evidence for the strong activity of HAA toward t(8;21) AML.

## Introduction

Acute myeloid leukemia (AML) with t(8;21) is considered a favorable cytogenetic subgroup, with a 3‐year survival of over 80% according to recent results from our group and others 1, 2, 3. Recently, homoharringtonine combined aclarubicin and cytarabine (HAA) has been demonstrated to achieve a high complete remission (CR) rate and provide a survival advantage in patients with acute myeloid leukemia (AML) 4, 5. According to a subgroup analysis, HAA showed significantly strong activity on t(8;21) and inv(16)/t(16;16), with an 89% CR rate after one cycle treatment. This supports previous results from some single‐center studies 6, 7. Moreover, as a salvage treatment, the HAA regimen has achieved a 90% CR rate in refractory/relapsed t(8;21) and inv(16)/t(16;16) AML 8. Our data showed that one cycle of HAA treatment can yield a 93% (28/30) CR rate in de novo t(8;21) AML patients 9. These results suggest that the HAA regimen is highly effective against t(8;21) AML.

To date, the underlying mechanisms by which HAA kills t(8;21) AML cells remain unclear. Homoharringtonine is an alkaloid derived from trees of the genus Cephalotaxus, having been used for more than 30 years to treat AML in China. The antileukemic effects of homoharringtonine are primarily based on the inhibition of protein synthesis, which induces differentiation, inhibits proliferation, and promotes apoptosis in leukemic cells 10, 11, 12, 13. Homoharringtonine also has a synergistic relationship with cytarabine and aclarubicin 11. Wang et al. confirmed that combining homoharringtonine with aclarubicin can result in synergistic cytotoxicity in THP‐1and Kasumi‐1 cells in vitro and in vivo 13. Combining homoharringtonine and aclarubicin simultaneously inhibited PI3K/AKT and WNT/β‐catenin signaling in AML cells 13. However, the mechanism of action of HAA in t(8;21)AML has yet to be explored. Therefore, we investigated the mechanism of the robust antileukemia effect of HAA on t(8;21)AML in vitro.

## Methods

### Cell lines and cell cultures

The AML cell lines SKNO‐1 and Kasumi‐1 were cultured in RPMI‐1640 (Gibco, Billings, MT, USA) supplemented with 10% fetal bovine serum (Gibco). All cell lines were grown at 37°C in a 5% CO_2_ atmosphere. Cell viability was assessed by triplicate counting of trypan blue dye‐excluding cells under light microscopy.

### Reagents and antibodies

Homoharringtonine was provided by Minsheng Pharmaceutical (Hangzhuo, China). Cytarabine was provided by Pfizer Pharmaceuticals (Nerviano, Italy). Aclarubicin was obtained from Wanle Pharmaceutical (Shenzhen, China). Fludarabine was provided by Pfizer Pharmaceuticals (Fuyang, China). Caspase‐3 and PARP antibodies were obtained from Abcam (Epitomics, Burlingame, CA, USA; Cambridge, UK). The antibody against ETO was obtained from Cell Signaling Technology (Danvers, USA).

### Growth inhibition assay

The inhibition of Kasumi‐1 and SKNO‐1 cell growth was evaluated by CCK‐8 assay (Dojindo Laboratories, Kumamoto, Japan). Briefly, 96‐well plates were seeded with 1 × 10^5^ cells per well and treated with various combinations of homoharringtonine, aclarubicin, and cytarabine. After 24 h or 48 h in culture, 10 μL of CCK‐8 solution was added to each well. The samples were incubated at 37°C for 4–6 h, and absorbance was measured at 450 nm using a microplate reader (Thermo Multiskan MK3, Waltham, MA, USA). Each sample was assayed with three replicates per assay, and cell‐line experiments were carried out in triplicate.

### Evaluation of apoptosis

Cells were treated with different combinations of reagents or equal volumes of DMSO for 12 h, harvested and washed twice with phosphate buffered saline (PBS; Gibco), resuspended in binding buffer and stained with Annexin V‐APC/7‐AAD according to the manufacturer's instructions (BD Pharmingen). A total of 5 μL of Annexin V‐FITC (BD Pharmingen, Torreyana Road, San Diego, CA, USA) and 5 μL of PI were added to the cell suspension. The data were expressed as the percentages of early apoptotic cells (Annexin V‐APC + /7‐AAD−) and late apoptotic cells (Annexin V‐APC + /7‐AAD+). Samples were analyzed on a FACSCalibur flow cytometer (BD Bioscience, San Jose, CA) equipped with CellQuest Pro software (BD Bioscience). For each sample, at least 1 × 10^5^ events were acquired for downstream analysis.

### Hoechst staining

SKNO‐1 and Kasumi‐1 cells were treated with different combinations of reagents or with equal volumes of DMSO for 12 h. Cells were then permeabilized with 0.5% Triton X‐100 for 30 min, washed with PBS, stained with 10 μg/mL Hoechst for 30 min, and washed with PBS. Nuclear morphology was observed immediately via fluorescence microscopy (BX51, Olympus, Tokyo, Japan).

### Western blot assay

Cells were harvested, ground in liquid nitrogen, lysed in lysis buffer (1% Triton X‐100, 0.015 mol/L NaCl, 1 mmol/L EDTA, 1 mmol/L phenylmethanesulfonyl fluoride (PMSF) and 10 g/mL each of leupeptin, aprotinin, and pepstatin A) and then incubated on ice for 30 min. Lysates were centrifuged at 12,000 *g* for 10 min at 4°C. Supernatants were mixed with one‐quarter volume of 4 × SDS sample buffer, boiled for 10 min and then separated by SDS‐PAGE on 10–12% gels. After electrophoresis, proteins were transferred to nitrocellulose (NC) membranes (Millipore Corporation, Bedford, MA, USA), blocked for 1 h with 5% non‐fat milk powder in TBST buffer (20 mmol/L Tris‐HCl, pH 7.6, 150 mmol/L NaCl, and 0.05% Tween‐20) and then incubated with primary antibodies at 1:1000 dilution overnight at 4°C. The membranes were washed three times with TBST and then incubated with secondary antibody at 1:5000 dilution for 1 h at RT. After extensive washing, proteins were visualized using an ECL‐Plus Kit (Thermo Scientific, Rockford, IL, USA), and blots were exposed to Kodak radiographic film.

### Statistical analysis

Data were expressed as the mean ± standard deviation (SD). The half‐maximal inhibitory concentrations (IC50) were calculated using GraphPad Prism (GraphPad Software, Inc., San Diego, CA, USA). The combination index (CI) was calculated using the Chou–Talalay method (Calcusyn software, Biosoft, San Diego, CA) to ascertain whether the effects of the drug combinations were synergistic (CI < 1), additive (CI = 1), or antagonistic (CI > 1). Categorical variables were compared using Fisher's exact test or the chi‐square test, and continuous variables were compared using the Wilcoxon rank sum test. *P* < 0.05 was considered statistically significant.

## Results

### Homoharringtonine combined with cytarabine and aclarubicin synergistically inhibited Kasumi‐1 and SKNO‐1 cell growth

**Figure 1:**
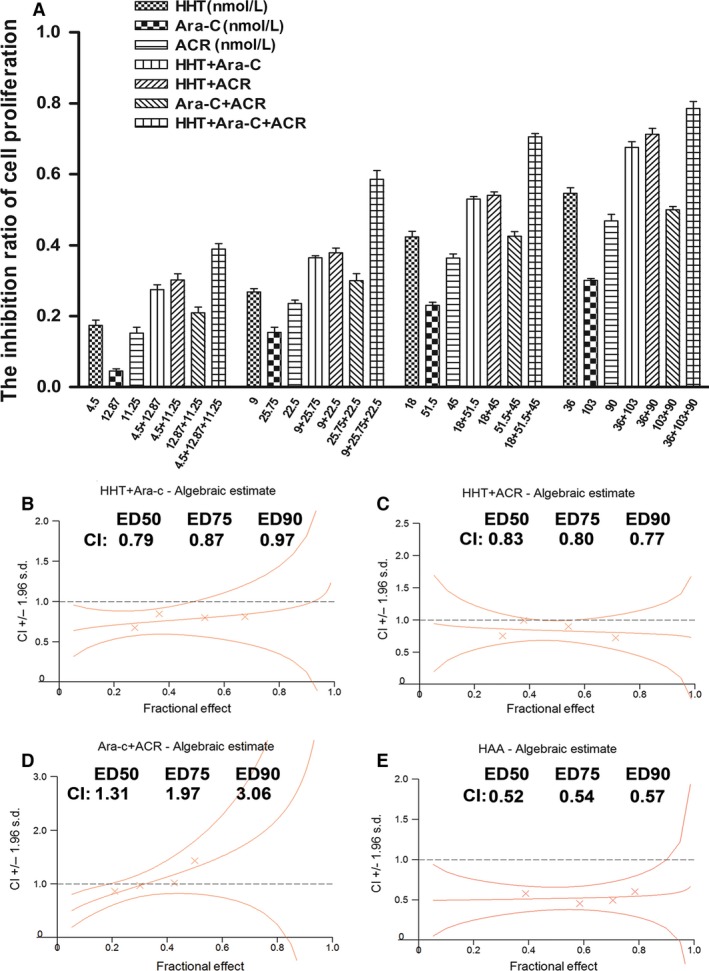
The growth inhibition and CI in Skno‐1 cells treated with different combinations. The rate of growth inhibition induced by homoharringtonine (HHT), cytarabine (Ara‐C), aclarubicin (ACR), HHT + Ara‐C, HHT + ACR, Ara‐C + ACR and HHT + Ara‐C + ACR in SKNO‐1 cells (A) for 24 h. CI values for HHT + Ara‐C combination treatments at a molar ratio of 1:2.86 in SKNO‐1 cells (B), HHT + ACR (1:2.5) (C), Ara‐C + ACR (1.14:1) (D), and HHT + Ara‐C + ACR (1:2.86:2.5) (E). We chose drug concentration gradients of HHT: 4.5, 9, 18, 36 nmol/L, Ara‐C: 12.87, 25.75, 51.5, 103 nmol/L, ACR: 11.25, 22.5, 45, 90 nmol/L. CI values for HHT + Ara‐C combination treatments at a molar ratio of 1:2.86 in SKNO‐1 cells (B), HHT + ACR combination treatments at a molar ratio of 1:2.5 (C), Ara‐C + ACR combination treatments at a molar ratio of 1.14:1 (D), and HHT + Ara‐C + ACR combination treatments at a molar ratio of 1:2.86:2.5 (E).

**Figure 2:**
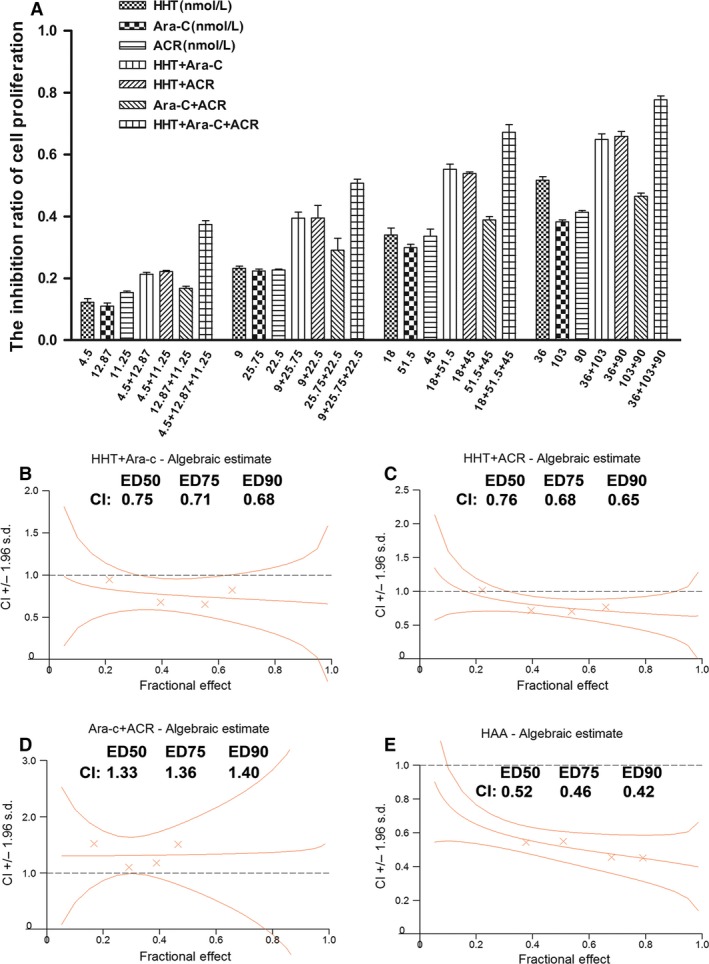
The growth inhibition and CI in Kasumi‐1 cells treated with different combinations. The rate of growth inhibition induced by homoharringtonine (HHT), cytarabine (Ara‐C), aclarubicin (ACR), HHT + Ara‐C, HHT + ACR, Ara‐C + ACR and HHT + Ara‐C + ACR in Kasumi‐1 cells for 24 h (A). CI values for HHT + Ara‐C combination treatments at a molar ratio of 1:2.86 in Kasumi‐1 cells (B), HHT + ACR (1:2.5) (C), Ara‐C + ACR (1.14:1) (D), and HHT + Ara‐C + ACR (1:2.86:2.5) (E). We chose drug concentration gradients of HHT: 4.5, 9, 18, 36 nmol/L, Ara‐C: 12.87, 25.75, 51.5, 103 nmol/L, ACR: 11.25, 22.5, 45, 90 nmol/L. CI values for HHT + Ara‐C combination treatments at a molar ratio of 1:2.86 in Kasumi‐1 cells (B), HHT + ACR combination treatments at a molar ratio of 1:2.5 (C), Ara‐C + ACR combination treatments at a molar ratio of 1.14:1 (D), and HHT + Ara‐C + ACR combination treatments at a molar ratio of 1:2.86:2.5 (E).

The following concentrations were used: homoharringtonine (4.5, 9, 18 and 36 nmol/L), cytarabine (12.87, 25.75, 51.5, and 103 nmol/L) and aclarubicin (11.25, 22.5, 45, and 90 nmol/L). DMSO was used as the control. The 24 h IC50 values of homoharringtonine, cytarabine, and aclarubicin for Kasumi‐1 and SKNO‐1 cells were (34.21 ± 3.51 nmol/L) and (28 ± 2.65 nmol/L), (195.07 ± 13.21 nmol/L) and (275.03 ± 8.57 nmol/L), and (148.9 ± 6.07 nmol/L) and (102.78 ± 13.78 nmol/L), respectively (*n* = 3). The 48 h IC50 values of homoharringtonine, cytarabine, and aclarubicin for Kasumi‐1 and SKNO‐1 cells were (16.90 ± 2.21 nmol/L) and (13.91 ± 1.72 nmol/L), (87.91 ± 14.84 nmol/L) and (144.27 ± 9.66 nmol/L), and (53.57 ± 7.21 nmol/L) and (51.37 ± 7.87 nmol/L) respectively (*n* = 3). The inhibition of Kasumi‐1 and SKNO‐1 cell growth was concentration‐dependent. The CCK‐8 assay showed dose‐dependent growth inhibition by homoharringtonine (HHT), cytarabine (Ara‐C) and aclarubicin (ACR) (*P* < 0.05). The growth‐inhibition effect of HAA on SKNO‐1 and Kasumi‐1 cells was stronger than that of any two‐drug combination at 24 h (Fig. 1A, 2B) (*P* < 0.001). Using Calcusyn 2.0, we found that HAA (1:2.86:2.5) also showed the strongest synergistic effect with CI values of (*X* and *Y*) for SKNO‐1 and Kasumi‐1 cells, respectively (Fig. 1B–E and 2B–E).

### Homoharringtonine combined with cytarabine and aclarubicin synergistically induced apoptosis in Kasumi‐1 and SKNO‐1 cells

**Figure 3:**
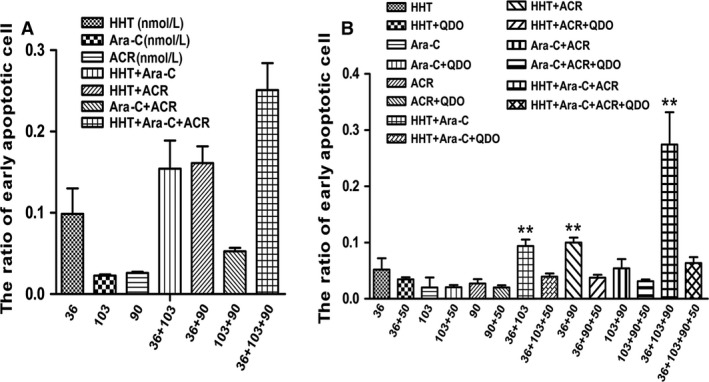
Apoptosis of SKNO‐1 and Kasumi‐1 cells detected by FCM. Apoptosis induced by homoharringtonine (HHT), cytarabine (Ara‐C), aclarubicin (ACR), HHT + Ara‐C, HHT + ACR, Ara‐C + ACR and HHT + Ara‐C + ACR in Kasumi‐1 (A), with or without 50 μmol/L caspase‐3 inhibitor Q‐DEVD‐OPh (QDO) in SKNO‐1 cells (B). Cells were cultured for 12 h and detected by FCM. **VS QDO group *P *< 0.01.

**Figure 4:**
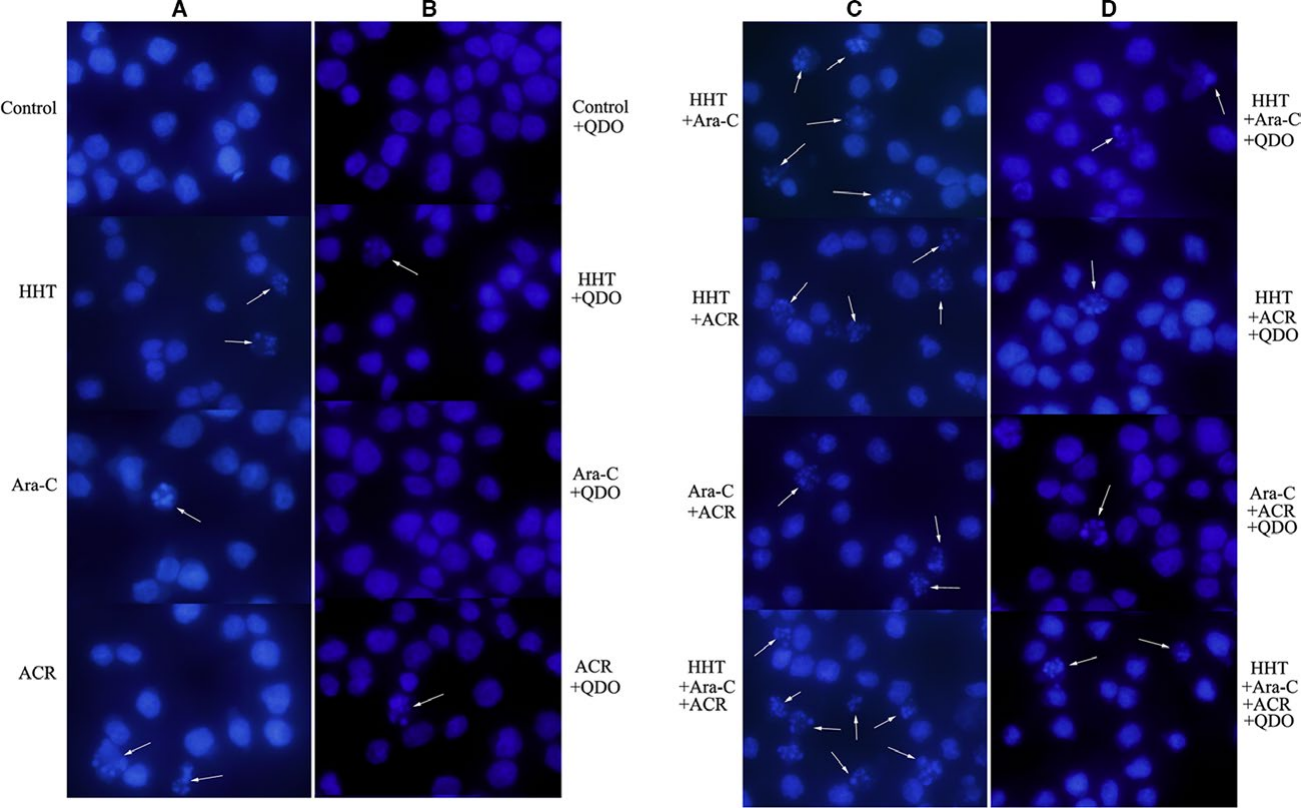
Caspase‐3 inhibitor Q‐DEVD‐OPh (QDO) inhibit apoptosis of Kasumi‐1 cells. Hoechst staining showed a higher number of condensed and/or fragmented nuclei in Kasumi‐1 cells when homoharringtonine (HHT) was combined with cytarabine (Ara‐C) and aclarubicin (ACR) (A, C), which were lower in the QDO groups (B, D) (combined with 50 μmol/L QDO).

The concentrations of homoharringtonine, cytarabine, and aclarubicin were 36, 103 and 90 nmol/L, respectively. SKNO‐1 and Kasumi‐1 cells treated with HAA had the highest percentage of apoptotic cells compared with any two‐drug combination or with the individual drugs (Fig. 3, *n* = 3). Moreover, pretreatment with 50 μmol/L of the caspase‐3 inhibitor Q‐DEVD‐OPh (QDO) for 12 h significantly suppressed HAA‐induced apoptosis. Hoechst staining also showed a higher number of condensed and/or fragmented nuclei in Kasumi‐1 cells treated with HAA compared with any individual drug or pairwise combination of drugs (Fig. 4, *n* = 3).

### Homoharringtonine combined with cytarabine and aclarubicin synergistically induced cleavage of the AML1‐ETO (AE) oncoprotein in Kasumi‐1 and SKNO‐1 cells

**Figure 5:**
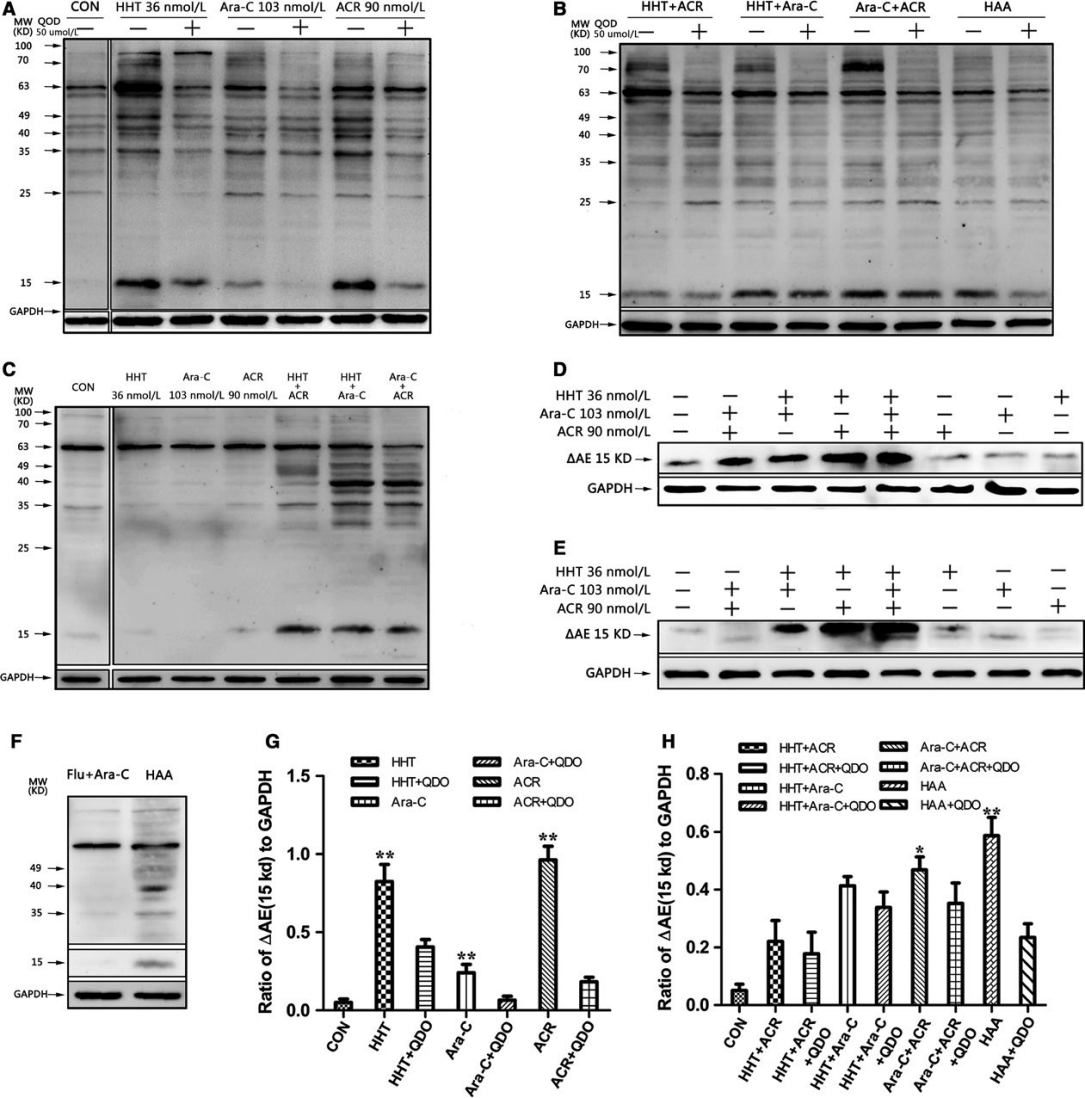
Different truncated AEs (ΔAEs) were found in SKNO‐1 and Kasumi‐1 cells. SKNO‐1 and Kasumi‐1 cells with or without 50 μmol/L caspase‐3 inhibitor Q‐DEVD‐OPh (QDO) were cultured with different combinations of 36 nmol/L homoharringtonine (HHT), 103 nmol/L cytarabine (Ara‐C) and 90 nmol/L aclarubicin (ACR) for 16 h. Expression of AML1‐ETO (AE) and AEs truncated AE (ΔAEs) was analysed via Western blotting. A total of six truncated ΔAEs were found in SKNO‐1 cells (A, B). A total of four truncated ΔAEs were found in Kasumi‐1 cells (C), including at 35 kD and 15 kD, which represent two novel ΔAEs. In addition, except the combination of 95 nmol/L fludarabine (Flu) and 103 nmol/L Ara‐C in Kasumi‐1 cells (F), a significant increase in the amount of 15‐kD ΔAE was observed in homoharringtonine combined with aclarubicin and cytarabine‐treated Kasumi‐1 cells (E) and SKNO‐1 cells (D, G, H). *VS QDO group *P *< 0.05; **VS QDO group *P *< 0.01.

The concentrations of homoharringtonine, cytarabine, and aclarubicin were 36, 103, and 90 nmol/L respectively. Cleavage of the AML1‐ETO (AE) oncoprotein in Kasumi‐1 and SKNO‐1 cells treated with HAA was higher than that observed for any individual drug or pairwise combination of drugs (Fig. 5, *n* = 3). Western blotting revealed six truncated AEs (ΔAEs) following treatment with the individual drugs or the various drug combinations, which was not markedly in the combination of fludarabine (95 nmol/L) adding cytarabine (103 nmol/L) (Fig. 5F).

**Figure 6:**
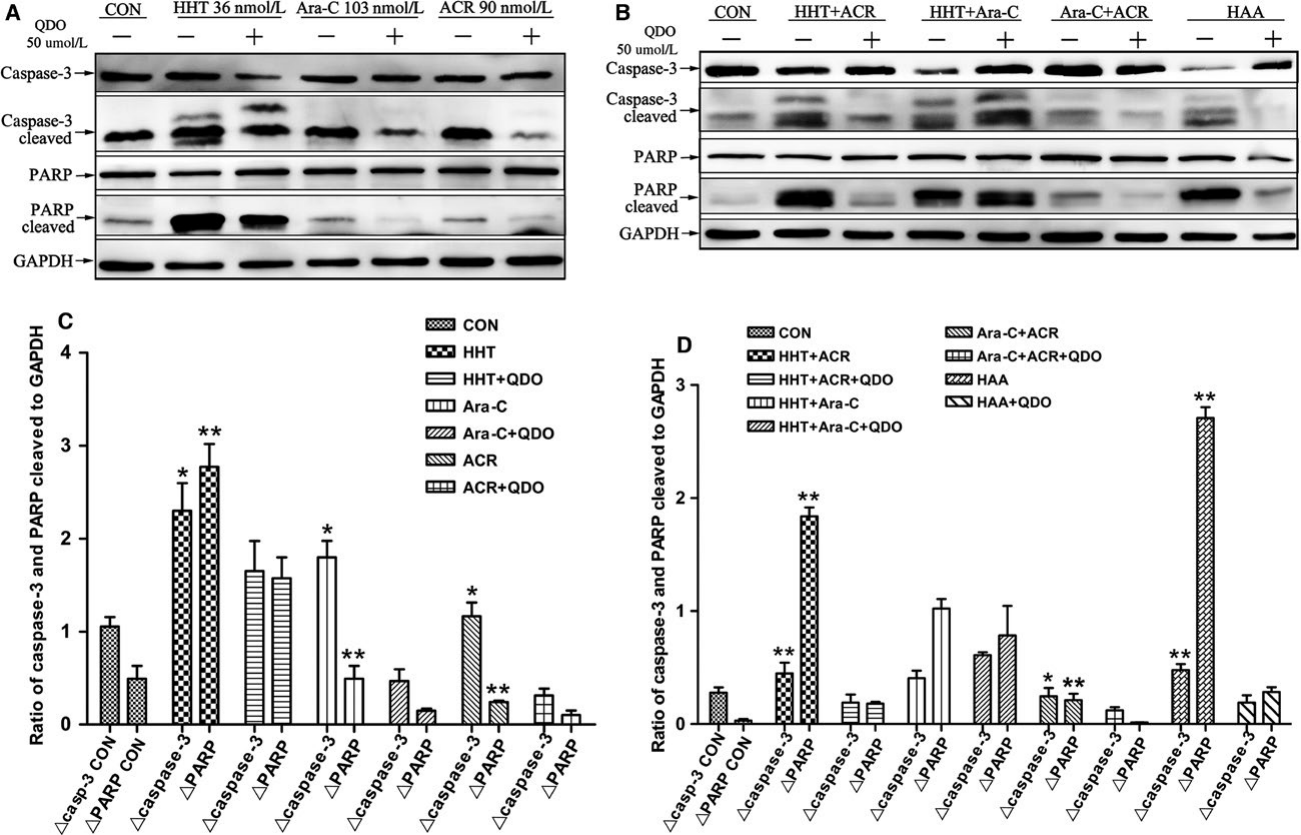
The expression of AE cleavage was abrogated by caspase‐3 inhibitor. Cells with or without 50 μmol/L caspase‐3 inhibitor Q‐DEVD‐OPh (QDO) were cultured for 16 h with 36 nmol/L homoharringtonine (HHT), 103 nmol/L cytarabine (Ara‐C) or 90 nmol/L aclarubicin (ACR) alone (A, C) and in various combinations (B, D). Western blot analyses of caspase‐3, cleaved caspase‐3 (Δcaspase‐3), PARP, and cleaved PARP (ΔPARP) protein expression in SKNO‐1 cells. Caspase‐3 activation and PARP degradation were significantly inhibited in each group except for the combination of homoharringtonine and aclarubicin, and AML1‐ETO (AE) cleavage was also markedly abrogated following pretreatment with 50 μmol/L caspase‐3 inhibitor QDO for 16 h. *VS QDO group *P* < 0.05; **VS QDO group *P *< 0.01.

Pretreatment with 50 μmol/L of the caspase‐3 inhibitor Q‐DEVD‐OPh (QDO) for 12 h abrogated AE cleavage and the generation of ΔAE (Fig. 5G, H, *n *= 3). We further analyzed the expression of caspase‐3, cleaved caspase‐3 (Δcaspase‐3), PARP, and cleaved PARP (ΔPARP). The activation of caspase‐3 and the degradation of PARP were significantly inhibited in each group except for that treated with the combination of homoharringtonine and aclarubicin (Fig. 6, *n* = 3).

## Discussion

Our study showed that, compared with single drugs or two‐drug combinations, HAA synergistically induced apoptosis in t(8;21) leukemia cells and triggered the caspase‐3‐mediated cleavage of the AML1‐ETO oncoprotein. This study provides in vitro evidence to explain the high efficacy of HAA treatment in t(8;21) AML patients.

The CI of HAA at the median effective dose (ED50) was 0.52 for SKNO‐1 cells, 0.52 for Kasumi‐1 cells. The high efficacy of HAA against t(8;21) AML may be due to the different mechanisms by which homoharringtonine, cytarabine, and aclarubicin kill leukemia cells. Homoharringtonine is an alkaloid derived from trees of the genus Cephalotaxus. The antileukemic effects of homoharringtonine are primarily based on the inhibition of protein synthesis, which induces differentiation, inhibits proliferation, and promotes apoptosis in leukemic cells 14, 15. The cytotoxicity of homoharringtonine is cell‐cycle specific, primarily affecting cells in the G1 and G2 phases 16. Moreover, homoharringtonine has been demonstrated to have significant synergistic effects with cytarabine 13, 17.

Aclarubicin, an alternative to daunorubicin, showed promising antileukemic efficacy in combination with cytarabine 18. Homoharringtonine, aclarubicin and cytarabine may also have no cross‐resistance, which may partially explain their potent antileukemia activity.

We wanted to elucidate the detailed events that occur during HAA treatment in leukemia cells with t(8;21). We found that HAA showed strongly inhibited growth and induced apoptosis in SKNO‐1 and Kasumi‐1 cells compared with individual drugs or two‐drug combinations. Our results support those of previous reports in which homoharringtonine, either alone or combined with aclarubicin, promoted apoptosis in Kasumi‐1, or primary AML cells.

The caspase‐3‐mediated cleavage of the AML1‐ETO oncoprotein was shown to be a major molecular mechanism of HAA activity in leukemia cells with t(8;21). When caspase‐3 action was blocked by an inhibitor, these cleaved fragments disappeared, strongly suggesting that caspase‐3 either directly or indirectly contributes to apoptosis‐related cleavage. Chen and coworkers also reported that the AML1‐ETO oncoprotein was degraded in parallel with caspase‐3 activation in apoptotic Kasumi‐1 cells induced by eriocalyxin B and oridonin 19, 20. Caspase‐3 activation, which is essential for leukemia cell apoptosis, leads to AML1‐ETO oncoprotein cleavage, resulting in the loss of ubiquitination sites and the generation of a 70‐kDa degradable form of AML1‐ETO (ΔAE) 20, 21. When SKNO‐1 and Kasumi‐1 were treated with mouse monoclonal anti‐Fas IgM antibody CH11, or were exposed to a germicidal lamp providing predominantly 254‐nm UV‐C light, 70, 49, 40, and 25 kDa were detected by anti‐ETO antibody during apoptosis 22. However, our study found that, when HAA was used, 35 and 15 kDa ΔAE were detected, and the generation of 15 kDa ΔAE could be inhibited by caspase‐3 inhibitor QDO (Fig. 5). ΔAE interacts with AML1‐ETO and interferes with the trans‐regulatory functions of the remaining AML1‐ETO oncoprotein 22. These results may partially explain why t(8;21) leukemia cells were more sensitive to HAA.

Our study has certain limitations. Apart from t(8;21) AML or primary cells from t(8;21) AML patients, no other types of AML cell lines were used in this study. This deficit may be improved in the future. We also have not included our clinical data on the efficacy of HAA treatment on t(8;21)AML patients, which are described separately.

In summary, our study suggests that HAA synergistically induces apoptosis in t(8;21) leukemia cells and triggers the caspase‐3‐mediated cleavage of the AML1‐ETO oncoprotein, thereby providing new insights into the biology and treatment of t(8;21)AML.
